# Retrospective analysis of urinary tract infections in long-term care facilities in Japan: Insights from physical examination-based diagnosis

**DOI:** 10.20407/fmj.2023-012

**Published:** 2023-11-29

**Authors:** Makoto Hasegawa, Yasuhiro Osugi, Yoshifumi Moriwaki, Yohei Doi

**Affiliations:** 1 Department of General Medicine, Toyota Regional Medical Center, Toyota, Aichi, Japan; 2 Department of Microbiology, Fujita Health University, School of Medicine, Toyoake, Aichi, Japan; 3 Department of Infectious Diseases, Fujita Health University, School of Medicine, Toyoake, Aichi, Japan

**Keywords:** Antimicrobial stewardship, Ceftriaxone, Febrile, Multidrug resistance, Urinary tract infection

## Abstract

**Objectives:**

Multidrug-resistant (MDR) bacterial infections are highly prevalent among long-term care facility (LTCF) residents, and are thus important targets for antimicrobial stewardship. Diagnoses of urinary tract infections (UTIs), which are associated with antimicrobial use in these facilities, are not always made by physicians. Past epidemiologic studies have included asymptomatic bacteriuria together with UTIs. The National Healthcare Safety Network has initiated a surveillance program to identify the causative organisms of UTIs in LTCF residents. In Japan, medical care for these residents is provided through in-person physician visits; however, limited related data are available. Therefore, we investigated the organisms causing UTIs and their drug susceptibility among LTCF residents in central Japan, and examined the prevalence of multidrug resistance, its risk factors, and correlations with clinical outcomes.

**Methods:**

We retrospectively evaluated clinical and urine culture data of LTCF residents with physician-diagnosed UTIs between April 1, 2019, and April 30, 2022.

**Results:**

The detection rate of multidrug-resistant organisms was high, with *Escherichia coli* being the most prevalent. Ceftriaxone was frequently used for initial therapy. The initial antimicrobial agents were significantly less active against MDR pathogens than non-MDR pathogens. Most residents continued to receive the initial agents regardless of culture results. Nonetheless, differences in the therapy duration, relapse and hospitalization rates, and death rate within 28 days between the multidrug-resistant and non–multidrug-resistant groups were not significant.

**Conclusions:**

Antimicrobial stewardship is essential for reducing antimicrobial use and selective pressure in LTCFs in Japan; however, more specific data are needed for its effective implementation.

## Introduction

Long-term care facilities (LTCFs) have come to play an increasingly important role in aging societies. In Japan, the number of LTCF residents in 2017 was 1.37 million, and is expected to increase to 1.78 million by 2025.^1^ The LTCF population is at high risk of developing infections, and up to 70% of residents receive antimicrobial therapy every year. Of these treatment courses, 40–75% are considered unnecessary or inappropriate.^2^ The use of broad-spectrum antimicrobials is common in this setting, prompting the emergence of multidrug-resistant (MDR) bacteria, both at the individual and institutional levels. Frequently hospitalized patients are also at risk of hospitalization-induced carriage of MDR bacteria.^3^ It has been reported that 20–40% of LTCF residents are colonized or infected with MDR bacteria.^4^ Multidrug resistance is a global health problem that limits options for antimicrobial therapy and increases the risk of treatment failure and mortality.^5^

As part of a multifaceted approach to tackling MDR bacteria, antimicrobial stewardship (AMS) programs have been proposed and implemented in many acute-care hospitals, and are aimed at preventing inappropriate antimicrobial use, reducing resistance rates, and improving patient outcomes. However, the application of AMS in LTCFs has lagged because of resource limitations and difficulties in data collection. Nonetheless, LTCFs remain a high priority for AMS application, given the considerable antimicrobial use in this setting and frequent transfer of patients to acute-care hospitals.^3^

Urinary tract infections (UTIs) are the most commonly diagnosed infections and, along with pneumonia, are a major cause of hospitalization for LTCF residents.^6^ LTCF residents with UTIs often have difficulty communicating urologic symptoms, which makes it challenging to differentiate symptomatic from asymptomatic bacteriuria. This situation is further complicated by limited access to diagnostic modalities and in-person examinations by physicians. Asymptomatic bacteriuria accounts for 75% of UTIs among LTCF residents and is a major cause of inappropriate antimicrobial use.^7^ Prophylactic administration and deviation from the recommended treatment periods are also common in the context of antimicrobial use.^8^ Furthermore, some reports have suggested that admission to an LTCF is an independent risk factor for bacteriuria due to MDR bacteria.^6,9^

The National Healthcare Safety Network (NHSN), using its own previously established criteria, reported antibiotic resistance rates as high as 36% for bacterial pathogens isolated from LTCF residents.^10^ The rates of multidrug resistance among *Klebsiella pneumoniae*, *Klebsiella oxytoca*, *Escherichia coli*, *Enterobacter* spp., and *Pseudomonas aeruginosa* reportedly range from 5.9% to 11.0%.^11^ The proportion of asymptomatic bacteriuria was also estimated to be high. Although efforts are underway to define and identify symptomatic UTIs in LTCFs for surveillance purposes,^12^ physical examination and symptom identification remain the key elements in making a clinical diagnosis. Therefore, there is a risk of underestimating the number of patients with UTIs in LTCFs, where many residents are older adults who have difficulty communicating.

Epidemiologic data are the foundation of effective AMS activities. However, because empirical treatment of UTIs without diagnostic urine testing is common in LTCFs in Japan, information regarding UTIs in this setting remains scarce. Our hospital is one of the largest training facilities for general practitioners in Japan and plays a key role in meeting the medical needs of LTCFs in central Japan. Physicians typically visit LTCFs, examine patients, and order urine testing in cases of suspected UTIs. Therefore, an accurate diagnosis of UTI, based on physical findings and epidemiologic data on the causative organism, can be expected.

We conducted a retrospective cohort study to investigate the causative organisms of UTIs and their susceptibility to antimicrobials, and the potential impact of multidrug resistance on clinical outcomes, among LTCF residents in Japan.

## Methods

### Study design

A retrospective cohort study was conducted among LTCF residents who were diagnosed with and treated for UTIs by physicians (general medicine residents and/or attendings in the Home Care Department of our hospital) who visited these facilities between April 1, 2019, and April 30, 2022. After excluding other diseases on the basis of physical examination results, residents with a fever (≥37.5°C) and an increased urinary leukocyte count (≥10 leukocytes /high-power field) at the time of the examination, and had been prescribed antimicrobial therapy, were included. The same patient could be included in the study more than once if more than 28 days had passed between diagnoses. Patients who were diagnosed with a UTI but did not meet the inclusion criteria were excluded. Additionally, LTCF residents who were on prophylactic antimicrobial therapy were excluded.

### Data collection and analysis

The following clinical information was collected: patient background (age, sex, and performance status [PS])^13^; status of the do-not-attempt-resuscitation (DNAR) order; clinical status at the time of diagnosis (e.g., altered mental status or hypotension); underlying diseases, including structural urinary disorders, history of hospitalization, and use of antimicrobial therapy in the previous 3 months; and relapse, hospitalization, and mortality rates within 28 days of the start of therapy (Table 1). Urine culture results were recorded, including the identified bacterial species and antimicrobial susceptibility testing results, which were interpreted on the basis of the criteria of the Clinical and Laboratory Standards Institute.^14^ Multidrug resistance was defined on the basis of published consensus guidelines.^15^ Differences in attributes between those infected with MDR pathogens (MDR group) and those not infected with MDR pathogens (non-MDR group) were compared using the Wilcoxon rank-sum test for continuous variables and Fisher’s exact test for categorical variables. Statistical significance was defined as a two-sided *P* value of <0.05.

### Ethics

This study was approved by the Clinical Ethics Committee of Toyota Regional Medical Center (approval number 21). Approval for an opt-out informed consent method using forms was given by the Clinical Ethics Committee of Toyota Regional Medical Center. Epidemiologic information in the electronic medical records was anonymized and assigned a number for use in correspondence.

## Results

### Study population

In all, 480 residents in the seven LTCFs covered by the department were examined by physicians during the study period. Sixty-seven UTI cases were confirmed, of which 35 met the inclusion criteria. After excluding five cases that represented relapse from the index UTI within the previous 28 days, 30 cases from 19 patients were included. Urine cultures were ordered, and the results were available for all cases.

### Patient characteristics

The overall patient population was characterized by high proportions of older women and individuals with low activities of daily living (ADL), of whom 33% were female. The median patient age was 79 years (standard deviation [SD], 15) and the median PS was 4 (SD, 1). Dementia and cerebrovascular disease were the most common underlying diseases, identified in 14 (47%) and 13 (43%) patients, respectively. Twenty-six cases (87%) had a DNAR order. Regarding UTI risk, the presence of an indwelling bladder catheter (N=22, 73%) and underlying diseases associated with dysuria (prostatic hyperplasia, neurogenic bladder, overactive bladder, and urolithiasis; N=17, 57%) were common. Twelve patients (40%) had been hospitalized within the previous 3 months.

### Causative pathogens

**Figure 1:**
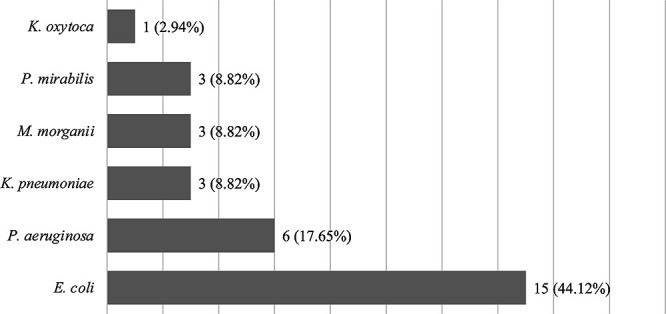
Bacterial strains isolated from urine cultures. *E. coli* was the most frequently isolated organism, followed by *P. aeruginosa.*

**Figure 2:**
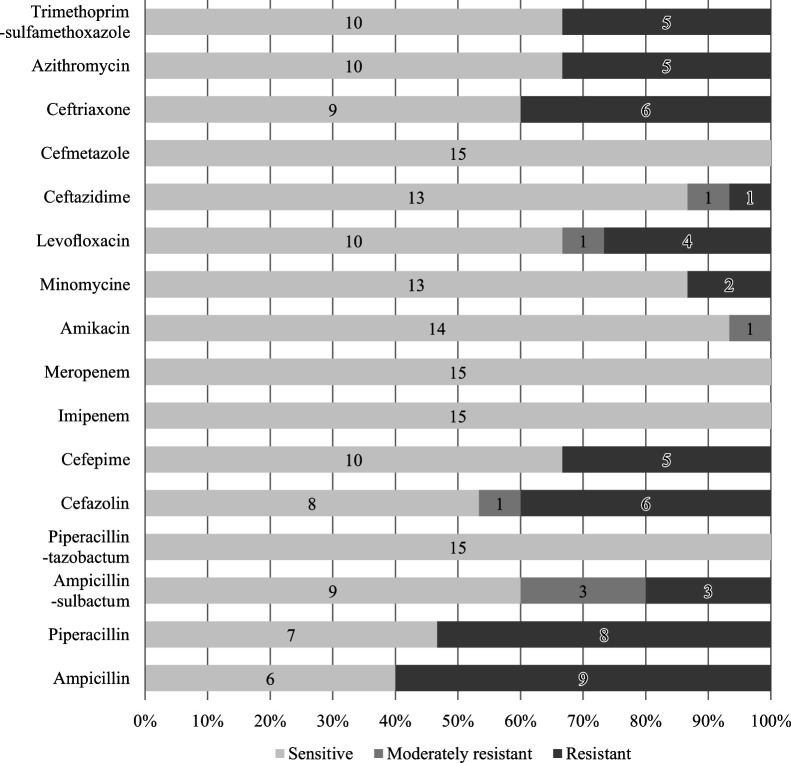
Antimicrobial susceptibility of *E. coli*, the most commonly isolated strain. The rate of non-susceptibility of *E. coli* to the tested antimicrobial agents was high. Forty percent of the organisms were resistant to third-generation cephalosporins, and 33% were resistant or moderately resistant to levofloxacin.

*E. coli* was the most common species identified in urine cultures (N=15, 44.1%), followed by *P. aeruginosa* (N=6, 17.7%) (Figure 1). Non-susceptibility of *E. coli* to the tested antimicrobial agents was high (Figure 2). Regarding the resistance to frequently used antimicrobial agents, 40% of the organisms were resistant to third-generation cephalosporins (9 susceptible and 6 resistant), and 33% were resistant or moderately resistant to levofloxacin (10 susceptible, 1 moderately resistant, and 4 resistant).

### Treatment and outcome

**Figure 3:**
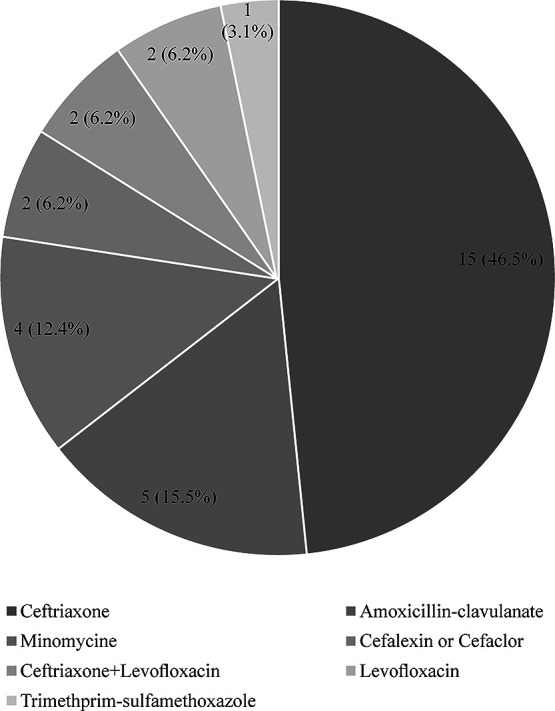
Antimicrobial agents used for the initial therapy of suspected UTI. Ceftriaxone was the most frequently used initial agent, followed by amoxicillin-clavulanate.

The antimicrobial agents used for initial therapy are shown in Figure 3. Ceftriaxone was the most frequently used initial agent (N=14, 47%), followed by amoxicillin-clavulanate (N=5, 17%). The characteristics of 16 (53%) cases of infection with MDR pathogens were compared with those of infection with non-MDR pathogens (N=14, 47%) (Table 1). The median age tended to be higher in the non-MDR group than in the MDR group (84 [SD, 15] vs. 74 [SD, 17] years; *P*=0.2), whereas the sex ratios (percentage of men) (N=4, 29% vs. N=6, 38%; *P*=0.7) and PS scores (4 [SD, 1] vs. 4 [SD, 0]; *P*=0.8) were similar. A decline in the PS from baseline was uncommon in both groups (N=1, 9% vs. N=1, 7%; *P*>0.9), and there were no cases of hypotension. The initial antimicrobial agent was far more likely to be effective in the MDR group than in the non-MDR group (N=11, 85% vs. N=3, 20%; *P*<0.001). Few patients received an appropriate change in antimicrobial agent based on culture results (N=2, 14% vs. N=1, 6.2%; *P*=0.6). There were no significant differences between the MDR and non-MDR groups in the duration of antimicrobial therapy (7 [SD, 1] vs. 9 [SD, 4] days; *P*=0.5), nor in rates of relapse (N=1, 8% vs. N=0, 0%; *P*=0.5), hospitalization, or death within 28 days (N=2, 14% vs. N=2, 12%; *P*>0.9).

## Discussion

MDR bacterial infections are highly prevalent in LTCFs, which are being increasingly recognized as important targets for AMS. While UTIs in LTCFs represent a major burden on antimicrobial use and resistance, the diagnosis of UTIs among LTCF residents is often based on exclusion, and is not necessarily made by a physician. Healthcare delivery in LTCFs differs from country to country. In many cases, physicians prescribe drugs based on reports from facility nurses that are delivered via phone call or e-mail. A physical examination is not always performed, which can lead to inappropriate antimicrobial administration.^16^ Limitations in both data collection and resources have slowed the application of AMS in LTCFs.

In Japan’s rapidly aging society, where over a quarter of the population is over 65 years of age, the situation remains unclear. LTCFs in Japan are home to large numbers of older people who require a high level of care for chronic conditions, such as dementia and cerebrovascular diseases. Although many facilities have on-site nurses, a characteristic of healthcare delivery in Japan is that physicians visit LTCFs to examine the patients in person, which allows for the diagnosis of UTIs based on the exclusion of other medical conditions. This study of LTCF residents in central Japan was aimed at exploring the causative organisms and drug susceptibility profiles of febrile UTIs diagnosed on the basis of physical examinations by general practitioners. The prevalence of multidrug resistance, its risk factors, and correlations with clinical outcomes were also examined.

Multidrug resistance accounted for 53% of infections with uropathogens in LTCFs in this study, a rate higher than that previously reported by the NHSN.^11^ Although a direct comparison may be difficult, the antimicrobial susceptibility of *E. coli* in this study suggested that resistance rates of uropathogens in LTCF residents may be as high as those in acute-care hospitals (Figure 2). The resistance rates in this study were comparable with those in large-scale epidemiologic data from hospital facilities in Japan.^17^ In particular, the high resistance rate of *E. coli* to third-generation cephalosporins (40%) is a concern. The prevalence of MDR bacteria among LTCF residents likely reflects a combination of factors, including frequent antimicrobial use, transmission among residents, and prolonged colonization. LTCFs in Japan are characterized by group activities, shared living quarters, and cognitive decline, all of which pose challenges to infection control and facilitate the transmission of resistant organisms.^18^ Furthermore, the patient care goals, medical resources, and staff proficiency in LTCFs differ greatly from those in acute-care hospitals, and education on infection control measures is often inadequate.^18^ The high detection rate of MDR organisms in our study raises concerns not only of inappropriate antimicrobial administration and spread of bacteria to and from hospitals, but also of transmission within institutions.

There was a trend toward higher dementia and cerebrovascular disease rates in the MDR group in this study, whereas a history of prior antimicrobial administration was more common in the non-MDR group, a finding contrary to those of previous studies.^4,9,19,20^ The previously reported risk factors for MDR carriage among LTCF patients, which include male sex, chronic wounds, use of healthcare-related devices, and history of antimicrobial administration, were not associated with multidrug resistance in our study.^20^ However, because our study was not powered to detect these associations, further studies are needed to determine whether these risk factors apply to LTCF residents in Japan. We anticipate that such studies could inform the selection of appropriate initial antimicrobials.

Most of the initial antimicrobials administered in the MDR group in this study were ineffective (Table 1); however, adjustments to achieve appropriate therapy based on culture results occurred only in a small number of cases. We suspect that difficulty in conducting a second visit, which is a prerequisite for medication order changes, prompted physicians to continue the initial therapy even when the pathogen showed resistance to the agent *in vitro*, as long as the patient was reported to be doing well clinically. Nevertheless, there were no significant intergroup differences in the duration of antimicrobial therapy, or rates of relapse, hospitalization, or death within 28 days. There are several possible explanations for this finding. One is that UTIs diagnosed on the basis of fever and pyuria, in the absence of an alternative fever source, may resolve spontaneously in many instances. In patients having catheter-associated UTIs with fever as the only symptom, fever may resolve after 24 hours of observation in two-thirds of patients; thus, some reports recommend a wait-and-see approach, administering an antimicrobial only if symptoms persist.^7^ In our study, none of the patients had severe conditions, such as hypotension or impaired consciousness. Thus, a careful follow-up without initial antimicrobial therapy may be possible for most UTI cases in LTCFs. Another explanation for our findings is that the urine concentration of an antimicrobial agent may be adequate to alleviate a UTI caused by a pathogen showing *in vitro *resistance to the agent. This is especially true for the penicillin, cephalosporin, and fluoroquinolone antimicrobial classes, which achieve urinary concentrations 100- to 1000-fold higher than those in blood owing to glomerular filtration and secretion in the collecting duct.^21^ Indeed, it has been suggested that the treatment of infections caused by strains that are categorized as resistant to the administered agent *in vitro* does not necessarily fail *in vivo*.^21^

Regarding antimicrobial selection, ceftriaxone was the most commonly administered agent (Figure 3). This finding differs from prescription data for other LTCFs in Japan, where macrolides and fluoroquinolones together accounted for the majority of prescriptions.^22^ There may be several reasons for this difference. Ceftriaxone can be administered even when patients have difficulty taking oral formulations. There are also increasing concerns about fluoroquinolones regarding their safety, increasing resistance, and inadvertent partial treatment of undiagnosed tuberculosis. Another unique factor that favored ceftriaxone use at the LTCFs in this study was that physicians at our facility could physically bring the drug for immediate use, whereas oral antibiotics had to be delivered from an off-site pharmacy post prescription, causing a lag in treatment initiation.

Our study has several limitations. As is often the case with LTCFs, the diagnosis of UTIs was made with consideration of the available resources, and certain diagnostic modalities, such as imaging, were inaccessible. However, diagnosis by exclusion of other diseases was likely to be performed relatively rigorously compared with that in similar settings in other countries, where physicians may not have direct involvement in the process. Furthermore, the timing of indwelling urinary catheter placement could not be ascertained among those with one catheter. Given that such catheters become colonized with bacteria,^23^ it is possible that some overdiagnosis may have occurred among those having asymptomatic bacteriuria with fever from another cause. Finally, the overall number of cases investigated was small, which limited the analysis to mostly descriptive data.

## Conclusions

Our findings support concerns regarding the high prevalence of UTIs due to MDR bacteria among LTCF residents. In addition to the unacceptably high rates of multidrug resistance, we found that antimicrobial prescriptions were seldom adjusted to appropriate agents on the basis of culture results indicating an MDR pathogen. However, no signs of adverse clinical outcomes due to inappropriate therapy were detected. Although structures and processes differ, the promotion of AMS is a common goal not only in acute-care hospitals but also in LTCFs. While the local epidemiology of infections in LTCFs, such as that reported here, supports AMS at the institutional level, a large-scale epidemiologic study of infections among LTCF residents, which could serve as a foundation for guiding AMS at LTCFs, is needed.

## Figures and Tables

**Table1:** Clinical information of patients included in the study

Characteristic	Overall, N=30^a^	Non-MDR, N=14^a^	MDR, N=16^a^	*P* value^b^
Age	79 years (15)	84 years (11)	74 years (17)	0.2
Sex; number of men	10 (33%)	4 (29%)	6 (38%)	0.7
Performance status	4 (1)	4 (1)	4 (0)	0.8
Unknown	1	0	1	
DNAR	26 (87%)	13 (93%)	13 (81%)	0.6
Altered consciousness	2 (8.0%)	1 (9.1%)	1 (7.1%)	>0.9
Unknown	5	3	2	
Low blood pressure	0 (0%)	0 (0%)	0 (0%)
Dementia	14 (47%)	3 (21%)	11 (69%)	0.01
Cardiovascular disease	1 (3.3%)	0 (0%)	1 (6.2%)	>0.9
Cerebrovascular disease	13 (43%)	3 (21%)	10 (62%)	0.024
Renal dysfunction	8 (27%)	5 (36%)	3 (19%)	0.4
Active malignancy	1 (3.3%)	1 (7.1%)	0 (0%)	0.5
Diabetes mellitus	5 (17%)	1 (7.1%)	4 (25%)	0.3
Indwelling bladder catheter	22 (73%)	10 (71%)	12 (75%)	>0.9
Diseases associated with dysuria	17 (57%)	9 (64%)	8 (50%)	0.4
Hospitalization up to 3 months ago	12 (40%)	7 (50%)	5 (31%)	0.3
Antimicrobials administered up to 3 months ago	10 (33%)	8 (57%)	2 (12%)	0.019
Effective initial antimicrobials	14 (50%)	11 (85%)	3 (20%)	<0.001
Unknown	2	1	1	
Appropriate antimicrobial changes based on culture results	3 (10%)	2 (14%)	1 (6.2%)	0.6
Duration of antimicrobial treatment	8 (3)	7 (1)	9 (4)	0.5
Unknown	3	2	1	
Relapse within 28 days	1 (3.8%)	1 (8.3%)	0 (0%)	0.5
Unknown	4	2	2	
Hospitalization or death within 28 days	4 (13%)	2 (14%)	2 (12%)	>0.9

^a^ Mean (standard deviation); n (%) ^b^ Wilcoxon rank-sum test; Fisher’s exact test; Pearson’s Chi-squared test

## Data Availability Statement

The datasets generated and/or analyzed during the current study are available from the corresponding author on reasonable request.
